# Targeted High-Throughput Sequencing Identifies Pathogenic Mutations in KCNQ4 in Two Large Chinese Families with Autosomal Dominant Hearing Loss

**DOI:** 10.1371/journal.pone.0103133

**Published:** 2014-08-12

**Authors:** Hongyang Wang, Yali Zhao, Yuting Yi, Yun Gao, Qiong Liu, Dayong Wang, Qian Li, Lan Lan, Na Li, Jing Guan, Zifang Yin, Bing Han, Feifan Zhao, Liang Zong, Wenping Xiong, Lan Yu, Lijie Song, Xin Yi, Ling Yang, Christine Petit, Qiuju Wang

**Affiliations:** 1 Institute of Otolaryngology, Chinese PLA General Hospital, Medical School of Chinese PLA, Beijing, China; 2 BGI-Tianjin, Tianjin, China; 3 Unité de Génétique et Physiologie de l'Audition, Institut Pasteur, Paris, France; 4 UMRS 1120, Institut National de la Santé et de la Recherche Médicale (INSERM), Paris, France; 5 Université Pierre et Marie Curie (Paris VI), Paris, France; 6 Collège de France, Paris, France; The University of Hong Kong, Hong Kong

## Abstract

Autosomal dominant non-syndromic hearing loss (ADNSHL) is highly heterogeneous, among them, *KCNQ4* is one of the most frequent disease-causing genes. More than twenty *KCNQ4* mutations have been reported, but none of them were detected in Chinese mainland families. In this study, we identified a novel *KCNQ4* mutation in a five generation Chinese family with 84 members and a known *KCNQ4* mutation in a six generation Chinese family with 66 members. Mutation screening of 30 genes for ADNSHL was performed in the probands from thirty large Chinese families with ADNSHL by targeted region capture and high-throughput sequencing. The candidate variants and the co-segregation of the phenotype were verified by polymerase chain reaction (PCR) amplification and Sanger sequencing in all ascertained family members. Then we identified a novel *KCNQ4* mutation p.W275R in exon 5 and a known *KCNQ4* mutation p.G285S in exon 6 in two large Chinese ADNSHL families segregating with post-lingual high frequency-involved and progressive sensorineural hearing loss. This is the first report of *KCNQ4* mutation in Chinese mainland families. *KCNQ4*, a member of voltage-gated potassium channel family, is likely to be a common gene in Chinese patients with ADNSHL. The results also support that the combination of targeted enrichment and high-throughput sequencing is a valuable molecular diagnostic tool for autosomal dominant hereditary deafness.

## Introduction

Hereditary hearing loss can be inherited in many patterns, such as autosomal dominant audosomal recessive, X-linked dominant, X-linked recessive, Y-linked pattern, among which ADNSHL has strikingly genetic heterogeneity. To date, more than 60 loci for ADNSHL have been mapped and only 30 corresponding genes have been identified (http://hereditaryhearingloss.org). During the past twenty years, linkage analysis and candidate gene sequencing has been proved to be a powerful tool to identify responsible genes for ADNSHL. However, limited number of samples in the clinical part, the large number of genes in the mapped region, and the large size of many genes restrained the application of this method. Recently, high-throughput sequencing, also known as next-generation sequencing (NGS) has been proved to be an ideal tool to decipher the genetic heterogeneity of deafness. More than ten deafness genes have been identified using NGS including *TPRN*, *GPSM2*, *HSD17B4*, *MASP1*, *CACAM1*, *HARS2*, *SMPX*, *DNMT1*, *ABHD12*, *TSPEAR*, *TNC* and *P2RX2* [1], [2], [3].

Among ADNSHL genes, *KCNQ4* (MIM*600101), one of the most frequent genes [4], was firstly identified as the causal gene for ADNSHL at DFNA2 by Kubisch and colleagues [5]. As a member of voltage-gated potassium channel family, *KCNQ4* plays a crucial role in potassium recycling in the inner ear. *KCNQ4* has six predicted transmembrane domains encoded by six exons (exon 2 to 7) and a P-loop between transmembrane domains S5 and S6. The P-loop domain forms a channel pore, containing a potassium ion-selective filter, whose function is eliminated by mutations in the pore region [5]. To date, 20 mutations in *KCNQ4* have been reported (Table 1) and it is identified as a common gene with a frequency up to 6.62% in ADNSHL in Japan, predicted to be the most prevalent gene responsible for Japanese ADNSHL patients [6]. However, these mutations have not been found in Chinese mainland populations before this study. Almost all reported cases showed a similar phenotype characterized by post-lingual, progressive, high-frequency hearing impairment (one mid-frequency predominant hearing loss caused by the p.V230E mutation was reported) [6].

**Table 1:** Overview of all *KCNQ4* mutations identified to date.

Mutation DNA	Protein	Exon	Origin	reference
c.211_223del13	p.Q71fs	1	Belguim	Coucke et al. (1999)
c.211delC	p.Q71fs	1	Japan	Kamada et al. (2006)
c.229_230insGC	P.H77fs	1	Japan	Naito, et al. (2013)
c.546C>G	p.F182L	4	Taiwan,	Su, et al. (2007);
			Japan	Naito, et al. (2013)
c.664_681del18	p.G215_220del6	4	Korea	Beak, et al. (2010)
c.689T>A	p.V230E	4	Japan	Naito, et al. (2013)
c.725G>A	p.W241X	5	USA	Hildebrand, et al. (2008)
c.778G>A	p.E260K	5	USA	Hildebrand, et al. (2008)
c.785A>T	p.D262V	5	USA	Hildebrand, et al. (2008)
c.821T>A	p.L274H	5	Neth	Van Hauwe, et al. (2000); De Heer, et al. (2011)
**c.823T>C**	**p.W275S**	**5**	**China**	**Present study**
c.827G>C	p.W276S	5	Neth, Japan	Coucke et al. (1999), Akita et al. (2001), Camp, et al. (2002), Topsakal, et al. (2005)
c.842T>C	p.L281S	6	USA	Talebizadeh, ea al. (1999)
c.853G>T	p.G285C	6	USA	Coucke et al. (1999)
**c.853G>A**	**p.G285S**	**6**	France,	Kubisch, et al. (1999)
			**China**	**Present study**
c.859G>C	p.G287R	6	USA	Arnett, et al. (2011)
c.871C>T	p.P291S	6	Japan	Naito, et al. (2013)
c.872C>T	p.P291L	6	Japan	Naito, et al. (2013)
c.886G>A	p.G296S	6	Spain	Mencia, et al. (2008)
c.891G>T	p.R297S	6	Japan	Naito, et al. (2013)
c.961G>A	p.G321S	7	Neth	Coucke et al. (1999)

Recently, we performed targeted capture and NGS to analyze a cohort of 30 hearing loss probands from Chinese families with ADNSHL (data not shown). Among these families, we identified a novel *KCNQ4* mutation p.W275R in exon 5 in family 025 and a known *KCNQ4* mutation p.G285S in exon 6 in family 727.

## Materials and Methods

### Ethics Statement

The study was approved by the Committee of Medical Ethics of Chinese People's Liberation Army (PLA) General Hospital. We obtained written informed consents from all the participants in this study. Written informed consents were obtained from the next of kin on the behalf of the minors/children participants involved in this study.

### Family Recruitment and Clinical Evaluations

**Figure 1:**
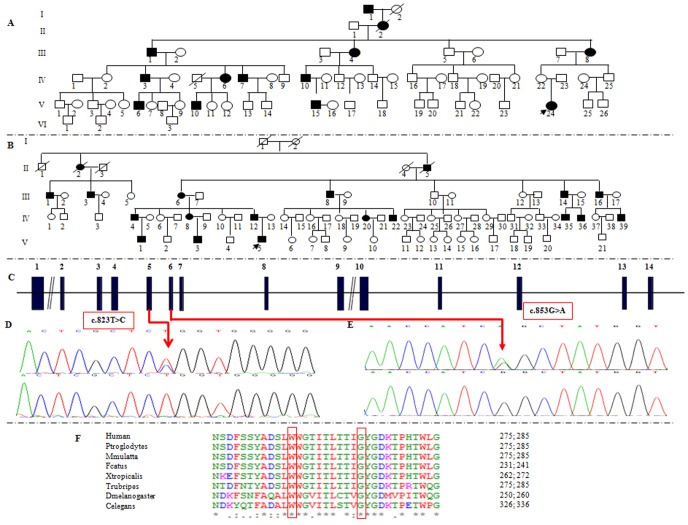
Pedigrees and mutation analysis of the two large Chinese families. (A) & (B) Pedigrees of family 025 and 727. Filled symbols for males (squares) and female (circles) represent affected individuals, and empty, unaffected individuals. (C) Structure of *KCNQ4* gene. *KCNQ4* gene has 14 exons. Mutations of c.823T>C (p.W275R) and c.853G>A (p.G285S) identified in *KCNQ4* are located in exon5 and exon6 respectively. (D) DNA sequence chromatograms showing the two heterozygous missense mutations c.823T>C and c.853G>A in affected individuals (upper panel) compared with the wild type controls (lower panel). (F) Conservation analysis shows that the Trp residue at 275 and the Gly residue at 285 in *KCNQ4* is conserved across human, Ptroglodytes, Mmulatta, Fcatus, Trubripes, Dmelanogaster, Celegans, Xtropicalis.

A six-generation family (025) with 66 members segregating ADNSHL and a five-generation family (727) with 84 members segregating ADNSHL were ascertained from the Department of Otolaryngology, Head and Neck Surgery, at the Institute of Otolaryngology of PLA, Chinese PLA General Hospital (Figure 1A, 1B). Either personal or family medical evidence of hearing loss, tinnitus, vestibular symptoms, use of aminoglycosides, and other clinical abnormalities of the participants were identified by a team of experienced doctors and audiologists. Audiometric evaluations included pure tone audiometry, auditory brainstem responses (ABR) and distortion product otoacoustic emissions (DPOAE). High resolution computed tomography (HRCT) was also performed on some subjects to verify whether the family members had other complications other than hearing disorders.

### Targeted capture and NGS

Genomic DNA (gDNA) was extracted from the whole blood samples using the Blood DNA kit (TIANGEN BIOTECH, Beijing, China), and 1 ug of purified gDNA fragmented to 200–300 base pairs using an ultrasonoscope (Covaris S2, Massachusetts, USA). End-repair, adenylation and adapter ligation were performed for library preparation following the Illumina's protocol. The same amount of library were pooled then hybridized to the customized capture array (NimbleGen, Roche) including exons, splicing sites and immediate flanking intron sequences of 29 genes for non-syndromic autosomal dominant hearing loss and TNC, a novel causative gene for ADNSHL identified in our previous research (Table S1). Sequencing was carried out on Illumina HiSeq2000 to generate paired end reads (90 bps at each end) [7].

Raw image files were processed by Illumina Pipeline (version 1.3.4) for base-calling with default parameters. Reads were aligned to NCBI37/hg19 assembly using the BWA (Burrows Wheeler Aligner). SNPs and indels (inserts and deletions) were detected using the GATK software [8].

### Sanger sequencing

After filtering against multiple databases, sanger sequencing was used in all available members from family 025 and 727 to determine whether the potential mutations in causative genes co-segregated with the disease phenotype in these families or not. Direct PCR products were sequenced using Bigdye terminator v3.1 cycle sequencing kits (Applied Biosystems. Foster City, CA) and analyzed using a ABI 3700XL Genetic Analyzer.

## Results

### Clinical description

**Figure 2:**
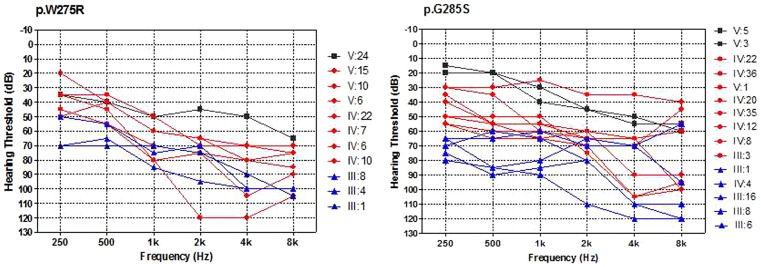
Overlapping audiograms from the better ear for each genotype. In the two cases of p.W275R and p.G285S, black colored audiograms were from the patients aged less than 20 years old, red colored audiograms were from individuals aged 20–49 years old and the blue audiograms were from the patients in their 50 s and over.

For family 727, a total of 41 family members, composed of 15 clinical affected and 26 unaffected individuals were ascertained in this study. While in family 025, 11 patients and 26 control individuals were ascertained. Age of onset in family 727 ranged from 5 to 30 years old, with average onset age 13.08 years old, while in 025 the onset age ranged from 2 to 30 years old, with average onset age 16.45 years old. For both two autosomal families, affected members showed a post-lingual, symmetrical, and bilateral non-syndromic sensorineural hearing loss. The hearing loss was initially presented as high frequencies with subsequent gradual progression to severe level involving all frequencies at later ages. Some patients had associated tinnitus, but no vestibular symptoms or signs were reported (Table 2, Table 3, Figure 1A, 1B, Figure 2). High resolution computed tomography (HRCT) of the temporal bone in the probands showed normal middle ears structure, including normal vestibular aqueduct and internal auditory canal. None of the affected members had a history of exposure to aminoglycosides, noise, or other causes that may account for the hearing impairment.

**Table 2:** Summary of clinical data for hearing impaired members in family 727.

Subject	Gender^a^	Age of test (year)	Age of onset (year)	PTA (dB HL)^b^	Hearing impairment^c^	Audiogram	Tinnitus
III:1	M	50	6	78.75	severe	downslope	+
III:3	M	44	10	72.50	severe	downslope	−
III:6	F	68	N/A	91.25	profound	flat	+
III:8	M	67	20	100.00	profound	downslope	+
III:16	M	54	15	66.25	moderately severe	flat	−
IV:4	M	50	30	75.00	severe	flat	+
IV:8	F	41	24	58.75	moderately severe	downslope	+
IV:12	M	34	17	57.50	moderately severe	flat	+
IV:20	F	25	5	70.00	moderately severe	flat	−
IV:22	M	21	6	61.25	moderately severe	flat	+
IV:35	M	25	10	73.75	severe	downslope	−
IV:36	M	22	10	62.50	moderately severe	flat	−
V:1	M	23	N/A	31.25	mild	downslope	N/A
V:3	M	13	11	38.75	mild	downslope	+
V:5	F	8	6	37.50	mild	downslope	+

a M, male; F, female.

b PTA, pure-tone air-conduction averages (0.5, 1, 2 and 4 kHz) for the better-hearing ear of affected subjects in family 727.

c Diagnosed at the time of test. The severity of hearing impairment was defined as mild (26–40 dB HL), moderate (41–55 dB HL), moderately severe (56–70 dB HL), severe (71–90 dB HL) and profound (>90 dB HL).

N/A, not available; +, positive finding; −, negative finding.

**Table 3:** Summary of clinical data for hearing impaired members in family 025.

Subject	Gender^a^	Age of test (year)	Age of onset (year)	PTA (dB HL)^b^	Hearing impairment^c^	Audiogram	Tinnitus^d^
III:1	M	76	15	76.25	severe	flat	+
III:4	F	72	20	86.25	severe	downslope	−
III:8	F	57	20	75	severe	downslope	+
IV:3	M	52	16	70	moderately severe	flat	+
IV:6	F	46	30	70	moderately severe	downslope	+
IV:7	M	39	25	66.25	moderately severe	downslope	−
IV:10	M	49	20	56.25	moderately severe	flat	−
V:6	M	25	17	61.25	moderately severe	downslope	−
V:10	M	25	15	56.25	moderately severe	downslope	−
V:15	M	24	10	58.75	moderately severe	flat	N/A
V:24	F	9	2	46.25	moderate	downslope	N/A

a M, male; F, female.

b PTA, pure-tone air-conduction averages (0.5, 1, 2 and 4 kHz) for the better-hearing ear of affected subjects in family 727.

c Diagnosed at the time of test. The severity of hearing impairment was defined as mild (26–40 dB HL), moderate (41–55 dB HL), moderately severe (56–70 dB HL), severe (71–90 dB HL) and profound (>90 dB HL).

d N/A, not available; +, positive finding; −, negative finding.

### Targeted high-throughput sequencing

Approximately 133K bp of exons and adjacent intronic regions of the 30 genes known to be responsible for ADNSHL were captured and sequenced. The average sequencing depth for target region is about 415×, and 99.44% of the average coverage for targeted region is more than 20×, which is satisfied with the requirements for calling SNPs and InDels (Table 4). For the proband of family 025, a total of 484 variants were identified, 47 of which were nonsynonymous variants, splice acceptor and donor site mutations and coding indels that were more likely to be pathogenic mutations; only 3 of the 47 variants were with the allele frequency which were less than 0.01 in dbSNP137, HapMap, 1000 human genome and local dataset; and a missense variant, c.823T>C (p.W275R) in exon5 of *KCNQ4* (NM_004700), predicted to be “Damaging”, “Probably Damaging”, “Deleterious”, “Disease_causing”, “Conserved” and “Conserved” by SIFT, Polyphen2, LRT, Mutation Taster, GERP++, and PhyloP respectively (Tables 5 and 6). For this site, 45% (114/254) reads supported for C vs. 55% (140/254) reads supported for T, which means it is a heterozygote (Het) (Figure S1). This indicated that this novel mutation may be the cause of the hearing loss in this Chinese family. In the proband of family 727, another missense mutation, c.853G>A (p.G285S) in *KCNQ4*, a previously reported mutation (rs28937588) was identified (Figure S2).

**Table 4:** Target region capture sequencing results.

Proband	Length of target region (bp)	Target Region Map Bases (Mbp)	Coverage (%)	Coverage at least 20× (%)	Mean Depth
025	132,789	54.21	99.71	99.49	408.23
727	132,789	56.11	99.71	99.38	422.59
**Average**	**132,789**	**55.16**	**99.71**	**99.44**	**415.41**

**Table 5:** Candidate genetic variants identified for the proband of family 025.

Filter process	NO. of Variants
All SNPs/InDels	484
Functional_variations	47
Genotype frequency in dbSNP137, HapMap, 1000 human genome dataset ≤0.01	8
Genotype frequency in local dataset≤0.01	3
Predicted to be deleterious by SIFT, Polyphen2, LRT and MutationTaster	1

**Table 6:** A novel variant in the proband of family 025.

Gene	NM No.	Nucleotide	Amino Acid	Zygosity	Prediction information					
					SIFT	Polyphen2	LRT	MutationTaster	GERP++	PhyloP
*KCNQ4*	NM_004700	c.823T>C	p.W275R	Het	Damaging	Probably Damaging	Deleterious	Disease_causing	Conserved	Conserved

### Mutation detection and analysis

Sanger sequencing confirmed the co-segregation of p.W275R and p.G285S with the disease phenotype in Family 025 and 727 respectively (Figure 1C and D and E). The two mutations were not detected in other 28 probands from Chinese families with ADNSHL. Both of the mutations occurred at highly conserved amino acids (Figure 1F), and are predicted to be deleterious by the SIFT, Polyphen2, LRT, Mutation Taster, GERP++, and PhyloP programs (IS IT NOT IDENTICAL TO WHAT HAVE MENTIONNED JUST ABOVE (Table 6). Based on these results and the phenotypes of these two families, we concluded that these two mutations in the *KCNQ4* are responsible for the hearing loss in the family 025 and 727.

## Discussion

As is known, mutations in gene *KCNQ4* have been associated with ADNSHL, recognized as one of the most frequent causes of ADNSHL, is characterized by post-lingual autosomal dominant non-syndromic progressive sensorineural hearing loss, first affected the high frequencies according to GeneReviews. In this present study, we identified a known mutation and a novel mutation in the P-loop region of the *KCNQ4* potassium channel which yielded dominant non-syndromic hearing loss by high-throughput sequencing as well as conventional genetic testing. It is noteworthy that this is the first report of heterozygous mutations in the *KCNQ4* genes as a cause of ADNSHL in Chinese mainland families. In family 727, the G285S mutation in exon6 affects the first glycine in the GYG signature sequence of K+ channel pores, which has been identified in three affected members of a small French family with DFNA2. This mutation exert a strong dominant-negative effect on the wild type and render heteromeric channels nonfunctional [5]. By using adenoviral delivery of *KCNQ4* channels carrying G285S, Holt JR and colleagues demonstrated that *KCNQ4* channels contributed to the M-like conductances: G_K,n_ of the cochlear outer hair cells and G_K,L_ of the vestibular type I hair cells [9]. As far as we know, this is the second report about this mutation. Recurrent mutations in *KCNQ4* also include c.211delC (p.Q71fs), c.821T>A (p.L274H), c.827G>C (p.W276S), having been reported in more than one family. Among these recurrent mutations, mutation W276S is a hot spot mutation in Belgian, Dutch and Japanese families [10]. Together with specific audiogram configuration, recurrent mutations may promote genetic testing for ADNSHL with a particular phenotype [6]. In family 025, we found a novel missense mutation W275R in exon 5 of KCNQ4. The mutation c.823T>C (W275R) is immediately adjacent to the previously reported mutation c.827G>C (W276S). These two adjacent tryptophan residues, located with the pore helix, are highly conserved across different potassium channel families and play a important role in K^+^ channel function, presumably holding the pore open at a correct diameter [11]. Mutations in these conserved tryptophan residues result in a complete loss of function of K^+^ channel [12]. Previous studies have demonstrated that the W276S mutation lead to a dramatic decrease in *KCNQ4* surface expression with strong dominant-negative effects on the wild type (WT) *KCNQ4* subunit. We presume that the W275R mutation may have the similar functional mechanism [13], [14].

It is noteworthy that this is the first report of *KCNQ4* mutation in Chinese mainland families. Before this report, there was a report of *KCNQ4* missense mutation P182L in a Taiwan family. The mutation locates in the S3 domain of *KCNQ4*. However, it is not conserved in all KCNQ family and unlikely to be pathogenic according to some prediction program, such as SIFT, Polyphen Phylop, LRT, etc. In this study, we found a P182L mutation in one of the 30 probands from ADNSHL families which did not co-segregate with the disease phenotype in the family members. It was also found in a Japanese control sample with normal hearing [4], [15].

To date, 16 missense mutations and 4 indels in *KCNQ4* have been reported (Table 1). Phenotype-genotype correlation of *KCNQ4* has been summarized, most of the patients with missense mutation are younger-onset and pure all-frequency hearing loss, while patients with deletion mutations are later-onset and pure high-frequency hearing loss, so the two families in this study are [16], [17]. *KCNQ4* is also predicted to be a candidate gene for age-related hearing loss (ARHI) since *KCNQ4* mutation families have similar pattern of hearing loss with ARHI, especially a unique pattern of hearing loss with striking resemblance to ARHI, in which only the high frequencies were progressively affected while the lower frequencies remained intact until an older age [18]. To investigate the association of *KCNQ4* with ARHI, Van Eyken et al. examined *KCNQ4* and detected a significant association between *KCNQ4* and ARHI in two independent Caucasian populations. All SNPs are located in the same 13-kb region in the middle of the *KCNQ4* gene, indicating that the pathogenic variants for ARHI may locate in this region [19].

*KCNQ4* is likely to be a common gene in Chinese ADNSHL: i) *KCNQ4* is one of the more frequent genes in ADNSHL in comparison to the other reported genes [4]; ii) In a large Japanese cohort, *KCNQ4* is found to be the most prevalent gene responsible for Japanese ADNSHL. In their study, 19 families with 7 different mutations were identified in 287 probands from ADNSHL families [6]. Mutations of *KCNQ4* were also found in other east Asian region, such as Korea and other Japanese population (Table 1). iii) Our group found a copy variation of 47 base pairs insertion or deletion in the exon2 and exon3 intron sequence, supposing to be a specific marker for the hearing loss of the pedigree [20]. Then we performed mutation screening of *KCNQ4* in 71 patients with high frequency hearing loss and 40 unaffected individuals of matched geographical ancestry, and found the deletion of the second intron 47 bp in 5 patients as well as 2 males with normal hearing, the insertion of 47 bp in 11 patients [21].

Despite an increasing number of pathogenic *KCNQ4* mutations have been identified, the molecular aetiology of DFNA2 still unknown. The missense mutations are believed to exert a dominant-negative effect by interfering with the normal channel subunit. The two deletions, c.211delC and c.211_223del13, are proposed to exert a pathogenic effect through haploinsufficiency [3]. In the mouse models, loss of *KCNQ4* function leads to progressive sensorineural hearing loss, paralleled by selective degeneration of outer hair cells and spiral ganglion neurons [22]. Among the missense mutations, L274H, W276S, L281S, G285S, G296S and G321S are loss of function mutations and eletrophysiological studies have shown that these mutations lead to loss of *KCNQ4* currents [5], [13], [22], [23], [24]. However, the molecular mechanisms about how these mutations lead the loss of *KCNQ4* currents remain unknown. Recently Gao YH et al. reported two mechanisms underlying DFNA2, the decreased cell surface expression detected by immunofluorescent microscopy and Western blot and the impaired conductance of *KCNQ4* demonstrated by electrophysiological studies [14]. Because of the restriction of the lack of the understanding of the molecular aetiology, no therapeutic methods to prevent progressive hearing loss are available for now. Further functional studies regarding mutations in these residues in *KCNQ4* may help clarify the molecular mechanism, which in turn, will facilitate informative genetic counseling, early diagnosis and even treatment of hearing impairment [25]. It is anticipated that future management of these genetic hearing disorders will be more targeted to the cellular processes involved and improve the likelihood of hearing recovery.

In conclusion, we have shown two mutations in Chinese ADNSHL families using targeted high-throughput sequencing. This is the first report of *KCNQ4* mutation in Chinese mainland families, providing more information for discovering the molecular mechanism of KCNQ4 mutation-induced hearing loss. The results also support that the combination of targeted capture and NGS is a valuable molecular diagnostic tool for autosomal dominant hereditary deafness.

## Supporting Information

Figure S1 **Reads of proband from family 025 (chr1 41285133).**
(TIF)

Figure S2 **Reads of proband from family 727 (chr1 41285565).**
(TIF)

Table S1 **The non-syndromic autosomal dominant genes captured.**
(DOC)

File S1 **Data S1–S4.** Data S1. Next generation sequencing results of proband of family 025 (including all SNP and INDEL results). Data S2. Next generation sequencing results of proband of family 727 (including all SNP and INDEL results). Data S3. Sanger sequencing results of family 025. This file shows the sanger sequencing results of 36 members ascertained in this study. Data S4. Sanger sequencing results of family 727. This file shows the sanger sequencing results of 41 members ascertained in this study.
(RAR)
