# Supplementary figures and images for: Targeted High-Throughput Sequencing Identifies Pathogenic Mutations in KCNQ4 in Two Large Chinese Families with Autosomal Dominant Hearing Loss
Source: PLoS One. 2014 Aug 12;9(8):e103133. doi: 10.1371/journal.pone.0103133 (PMC4130520; doi:10.1371/journal.pone.0103133)

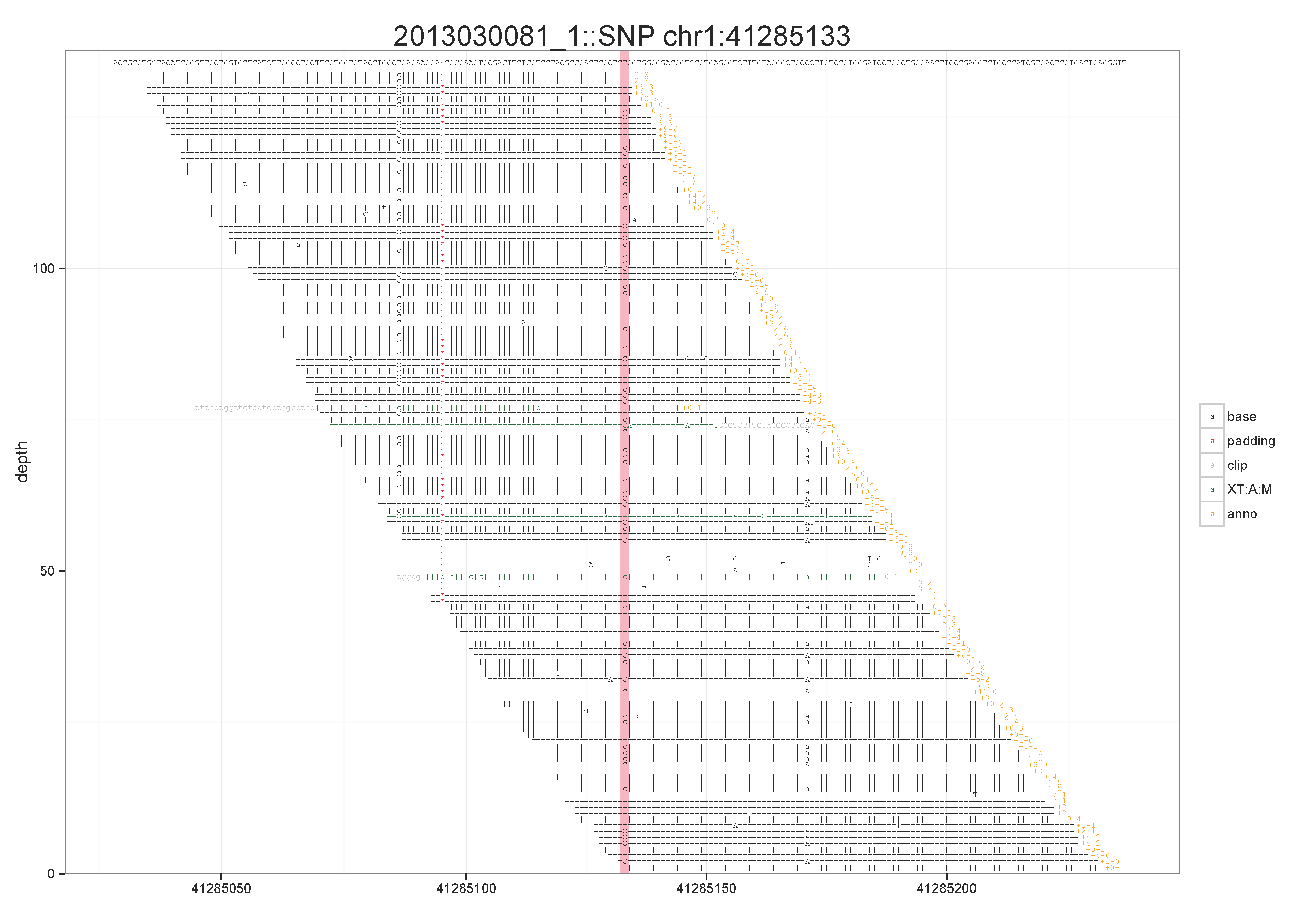

Supplement: Figure S1 — Reads of proband from family 025 (chr1 41285133). (TIF) [file pone.0103133.s001.tif]

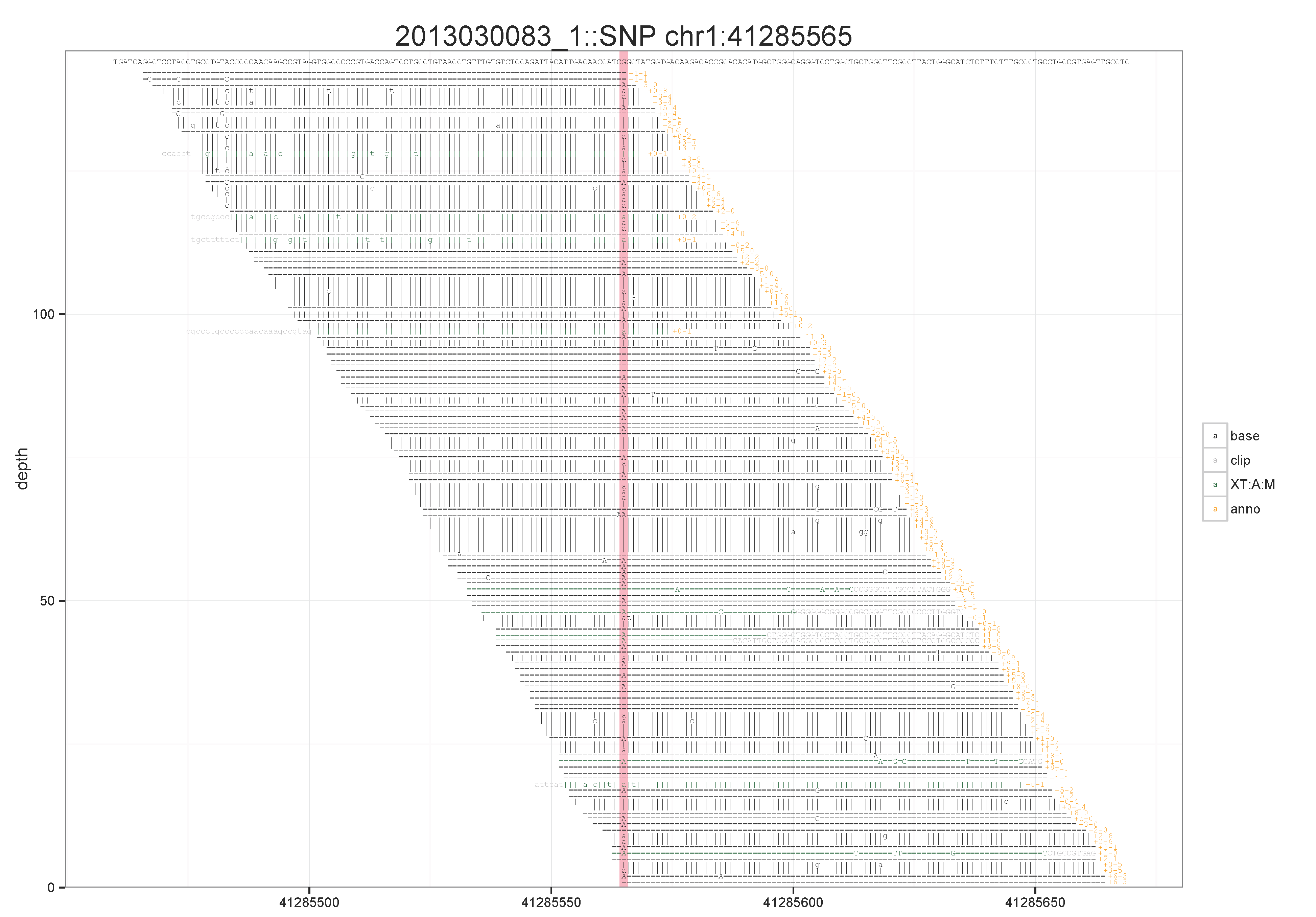

Supplement: Figure S2 — Reads of proband from family 727 (chr1 41285565). (TIF) [file pone.0103133.s002.tif]
