# Supplementary material for: Targeted High-Throughput Sequencing Identifies Pathogenic Mutations in KCNQ4 in Two Large Chinese Families with Autosomal Dominant Hearing Loss
Source: PLoS One. 2014 Aug 12;9(8):e103133. doi: 10.1371/journal.pone.0103133 (PMC4130520; doi:10.1371/journal.pone.0103133)
Supplement: Table S1 — The non-syndromic autosomal dominant genes captured. (DOC) [file pone.0103133.s003.doc]

**Table S1 The nonsydromic autosomal dominant genes captured**

| **Gene name** | **Chromosomal locus** | **Group** | **Locus of recessive NSHL** | **Locus of dominant NSHL** | **Human syndrome** | **Number of captured exons** | **Number of captured bp** |
| --- | --- | --- | --- | --- | --- | --- | --- |
| *ACTG1* | 17q25.3 | human NSHL |  | DFNA20/26 |  | 5 | 2,403 |
| *CCDC50* | 3q28 | human NSHL |  | DFNA44 |  | 12 | 9,920 |
| *COCH* | 14q12 | human NSHL |  | DFNA9 |  | 12 | 3,995 |
| *COL11A2* | 6p21.32 | human NSHL and SHL | DFNB53 | DFNA13 | Stickler, STL3 | 66 | 11,951 |
| *CRYM* | 16p12.2 | human NSHL |  | DFNA- |  | 12 | 2,640 |
| *DFNA5* | 7p15.3 | human NSHL |  | DFNA5 |  | 11 | 3,455 |
| *DIAPH1* | 5q31.3 | human NSHL |  | DFNA1 |  | 28 | 8,058 |
| *DSPP* | 4q22.1 | human NSHL |  | DFNA39 |  | 5 | 4,734 |
| *EYA4* | 6q23.2 | human NSHL |  | DFNA10 |  | 21 | 7,772 |
| *GJB2* | 13q12.11 | human NSHL | DFNB1 | DFNA3 |  | 2 | 2,493 |
| *GJB3* | 1p34.3 | human NSHL |  | DFNA2 |  | 3 | 4,644 |
| *GJB6* | 13q12.11 | human NSHL | DFNB1B | DFNA3B |  | 5 | 2,971 |
| *GRHL2 / TFCP2L3* | 8q22.3 | human NSHL |  | DFNA28 |  | 16 | 6,523 |
| *KCNQ4* | 1p34.2 | human NSHL |  | DFNA2 |  | 14 | 3,469 |
| *MIR96* | 7q32.2 | human NSHL |  | DFNA50 |  | 1 | 156 |
| *MYH14* | 19q13.33 | human NSHL |  | DFNA4 |  | 43 | 10,395 |
| *MYH9* | 22q12.3 | human NSHL |  | DFNA17 |  | 41 | 10,825 |
| *MYO1A* | 12q13.3 | human NSHL |  | DFNA48 |  | 29 | 5,888 |
| *MYO6* | 6q14.1 | human NSHL | DFNB37 | DFNA22 |  | 35 | 11,497 |
| *MYO7A* | 11q13.5 | human NSHL and SHL | DFNB2 | DFNA11 | Usher, USH1B | 50 | 11,709 |
| *POU4F3* | 5q31 | human NSHL |  | DFNA15 |  | 2 | 1,344 |
| *SLC17A8* | 12q23.1 | human NSHL |  | DFNA25 |  | 12 | 4,955 |
| *TECTA* | 11q23.3 | human NSHL | DFNB21 | DFNA8/12 |  | 23 | 8,331 |
| *TJP2* | 9q21.11 | human NSHL |  | DFNA51 |  | 25 | 8,642 |
| *TMC1* | 9q21.13 | human NSHL | DFNB11 | DFNA36 |  | 24 | 5,145 |
| *WFS1* | 4p16.1 | human NSHL |  | DFNA6/14 |  | 9 | 4,801 |
| *CEACAM16* | 19q13.31 | human NSHL |  | DFNA4 |  | 8 | 1,995 |
| *SMAC/DIABLO* | 12q24.31 | human NSHL |  | DFNA64 |  |  |  |
| *DIAPH3* | 13q21.2 | human NSHL |  | AUNA1 |  |  |  |
| *TNC* | 9q32-q34 | included in DFNA56 |  |  |  |  |  |
